# The acyl chains of phosphoinositide PIP3 alter the structure and function of nuclear receptor steroidogenic factor-1

**DOI:** 10.1016/j.jlr.2021.100081

**Published:** 2021-04-29

**Authors:** Jamal M. Bryant, M. Merced Malabanan, Boden H. Vanderloop, Charles M. Nichols, Zeinab Haratipour, Katrina T. Poon, Stacy D. Sherrod, John A. McLean, Raymond D. Blind

**Affiliations:** 1 Department of Pharmacology, Vanderbilt University School of Medicine, Nashville, TN, USA; 2 Department of Medicine, Division of Diabetes, Endocrinology and Metabolism, Vanderbilt University Medical Center, Nashville, TN, USA; 3 Vanderbilt Diabetes Research and Training Center, Vanderbilt University Medical Center, Nashville, TN, USA; 4 Center for Innovative Technology and Department of Chemistry, Vanderbilt University, Nashville, TN, USA; 5 Genomic Medicine Training Program, Vanderbilt University School of Medicine, Nashville, TN, USA; 6 Department of Biochemistry, Vanderbilt University School of Medicine, Nashville, TN, USA; 7 Center for Structural Biology, Vanderbilt University School of Medicine, Nashville, TN, USA

**Keywords:** nuclear PIP3, PtdIns(3,4,5)P3, PtdIns(4,5)P2, nuclear receptors, X-ray crystallography, AF2, activation function 2, DBD, DNA-binding domain, LBD, ligand-binding domain, MRM, multiple reaction monitoring, PGC1α, Peroxisome proliferator-activated receptor gamma coactivator 1-alpha, SF-1, Steroidogenic Factor-1

## Abstract

Nuclear receptors are transcription factors that bind lipids, an event that induces a structural conformation of the receptor that favors interaction with transcriptional coactivators. The nuclear receptor steroidogenic factor-1 (SF-1, *NR5A1*) binds the signaling phosphoinositides PI(4,5)P2 (PIP2) and PI(3,4,5)P3 (PIP3), and our previous crystal structures showed how the phosphoinositide headgroups regulate SF-1 function. However, what role the acyl chains play in regulating SF-1 structure remains unaddressed. Here, we used X-ray crystallography with *in vitro* binding and functional assays to examine how the acyl chains of PIP3 regulate human SF-1 ligand-binding domain structure and function. Altering acyl chain length and unsaturation regulates apparent binding of all tested phosphoinositides to SF-1. Mass spectrometry–based lipidomics data suggest C16 and C18 phospholipids preferentially associate with SF-1 expressed ectopically in bacteria. We then solved the 2.5 Å crystal structure of SF-1 bound to dioleoyl PIP3(18:1/18:1) to compare it with a matched structure of SF-1 bound to dipalmitoyl PIP3(16:0/16:0). The dioleoyl-bound structure was severely disordered in a specific SF-1 region associated with pathogenic human polymorphisms and within the coactivator-binding region critical for SF-1 function while inducing increased sensitivity to protease digestion in solution. Validating these structural observations, *in vitro* functional studies showed dioleoyl PIP3 induced 6-fold poorer affinity of a peroxisome proliferator-activated receptor gamma coactivator 1-alpha coactivator peptide for SF-1 compared with dipalmitoyl PIP3. Together, these data suggest the chemical nature of the phosphoinositide acyl chains controls the ordered state of specific, clinically important structural regions in SF-1, regulating SF-1 function *in vitro*.

Nuclear receptors are a superfamily of DNA-binding transcription factors regulated by lipid ligands such as cholesterol-based steroids (1, 2), fatty acids (3, 4), and phospholipids (5) among other hydrophobic molecules such as xenobiotics (6) and heme (7). Generally, nuclear receptors have important functions in metabolism, development, homeostasis, signaling, and reproduction in metazoans (8, 9). One of the 48-member nuclear receptor superfamily in humans is steroidogenic factor-1 (SF-1, *NR5A1*), which plays an essential role in development and adult function of steroidogenic tissue (10). SF-1 is required for proper regulation of gene expression (11, 12, 13) and is essential for sexual determination and development of the adrenals, gonads, and hypothalamus in mammals (14). SF-1 is also a well-validated target for drug design efforts seeking to develop therapeutics for the treatment of adrenocortical carcinoma and endometriosis. Thus, understanding how this nuclear receptor is regulated can provide insight into several pathologies and physiological processes in humans.

SF-1 binds many different classes of phospholipids with varying chemical composition of the headgroup and acyl chains. Ectopically expressed SF-1 in bacteria co-purifies and co-crystallizes with phosphatidylethanolamines and phosphatidylglycerols (15). SF-1 has also been co-crystallized with phosphatidylcholine (16), whereas sphingosine was identified by mass spectrometry as specifically associated with SF-1 in mammalian cells (17), and sphingosine metabolic enzymes can regulate SF-1 activity (18). There is also considerable evidence suggesting phosphoinositides as regulatory ligands for SF-1 (19). Despite the wide variety of phospholipid species that interact with SF-1, these lipids all share a common 1:1 stoichiometry, interacting within the same ligand-binding pocket at the C-terminal ligand binding domain (LBD) of SF-1, as shown by several X-ray crystal structures of the human and mouse orthologs of SF-1 (20). These structures collectively show the acyl chains to be buried deep within the hydrophobic core of the SF-1 LBD, whereas the headgroups are largely solvent exposed, particularly true of the phosphoinositides PIP2 and PIP3 (21). Thus, a wide variety of phospholipids bind SF-1 at the same binding site and have potential to regulate SF-1 activity.

Of all phospholipids that bind SF-1, the phosphoinositides bearing PI(4,5,)P2 (PIP2) and PI(3,4,5)P3 (PIP3) headgroups are among the most well characterized. Although sphingolipids have been identified as specifically associated with SF-1 biochemically purified from cell lines (17), there are no structures of SF-1 bound to any sphingolipid, and an endogenous ligand for SF-1 from mammalian tissue has yet to be identified. Phosphoinositides were suggested as endogenous ligands over 15 years ago (15), were among the first phospholipids to be tested for interaction with SF-1 (15), and bind with high affinity (21). The technical difficulties in detecting phosphoinositides by mass spectrometry remain a barrier to untargeted lipidomic approaches for identifying phosphoinositides unequivocally from mammalian cells and tissues using mass spectrometry (22). Though not to the exclusion of other lipids (17, 18, 23), there are several lines of evidence suggesting that the phosphoinositide PIP3 is a regulatory ligand for SF-1. Crystallographic studies showed atomic resolution details of how the PIP3 headgroup forms part of a novel regulatory surface on SF-1, which genetic studies demonstrated is required for full SF-1 function (21). Increased cellular levels of PIP3 correlate with increased SF-1 function in mammalian cells (24). The worm ortholog of SF-1 binds PIP3 in vitro and regulates phospholipid metabolism in vivo (25). Genetically or chemically inhibiting the nuclear inositol polyphosphate multikinase (26, 27, 28) which generates PIP3 regulates SF-1 function in human cells, whereas simply decreasing total cellular PIP3 by inhibiting the p110-class of PI3 kinases does not have similar effects (29, 30). Dipalmitoyl PIP3 has nanomolar affinity for SF-1, among the highest affinity of all phospholipids tested in previous work (21). Dipalmitoyl PIP3 also slightly enhances interaction of SF-1 with a peptide representing the peroxisome proliferator-activated receptor gamma coactivator 1-alpha (PGC1α) transcriptional coactivator in vitro (21), also suggesting PIP3 can enhance SF-1 transcriptional activity (30, 31). Perhaps, the best evidence that SF-1 binds phosphoinositides in living cells comes from immunoprecipitated SF-1 from human cells, which contains PIP2 as detected by ^32^P radiolabeling by in vitro kinase reactions, and PIP2 associated with SF-1 was estimated to occupy about half the SF-1 sites in these studies (29). Thus, a large body of growing evidence supports a model wherein PIP3 binds and activates SF-1, and the PIP3 headgroup participates in that activation.

While PIP3 headgroup-mediated effects on SF-1 activation are well supported by several direct tests in previous work, what effect the acyl chains have on SF-1 structure has not been directly addressed. The closest comparison that might indicate how the acyl chains alter SF-1 structure comes from comparing the crystal structures of SF-1 bound to PE(16:0/16:0) and a co-repressor peptide (PDB:1YP0) (32) versus SF-1 bound to PE(16:1-18:1) and a different co-activator peptide (PDB:1YOW) (15). Although these models show differences in SF-1 structure, the different peptides make assigning any affect to acyl chain composition difficult. Still, these and other structures (16) remain consistent with models in which the acyl chains might regulate SF-1 structure and thus the ability of SF-1 to interact with transcriptional coactivators.

Here, we directly address the question of how the acyl chains of phosphoinositides regulate the structure and function of SF-1 for the first time, using a new protein-sequence and headgroup-matched crystal structure, combined with in vitro binding assays and additional functional assays. The data show the acyl chains of PIP3 regulate SF-1 structure and function in vitro. Our data suggest that longer and more complex acyl chains induce structural disorder in SF-1 that is detectable by X-ray crystallography, occurring in regions of SF-1 previously shown to be critical for function in human patients (33, 34, 35, 36). Although changes to any crystal structure could be because of crystallographic artifacts, the changes we observe directly correlated with predicted changes to SF-1 using in vitro functional assays. Together, these data suggest the PIP3 acyl changes can regulate SF-1, directly addressing the role of the phosphoinositide acyl chains in regulating SF-1 structure and function.

## Materials and Methods

### Phosphoinositide reagents

Chemically synthesized PIP3(18:1(9Z)/18:1(9Z)) (1,2-di-(9Z-octadecenoyl)-sn-glycero-3-[phosphoinositol-3,4,5-trisphosphate]) was from Avanti Polar Lipids (Product number 850156, Alabaster, AL), and chemically synthesized PIP3(16:0/16:0) (1,2-dipalmitoyl-sn-glycero-3-[phosphoinositol-3,4,5-triphophosate]) was from Cayman Chemical Company (Ann Arbor, MI). All other phosphoinositides were purchased from Avanti. All lipids were stored lyophilized, in the dark at −20°C under vacuum.

### Protein expression and purification

The human SF-1 (*NR5A1*, Accession NP_004950.2) DNA plasmid construct, all protein and peptide sequences, and purification procedure used here were identical to our previous crystallographic analysis of human SF-1 LBD bound to dipalmitoyl PIP3(16:0/16:0) (21), except that dioleoyl PIP3(18:1(9Z)/18:1(9Z)) was complexed with SF-1. Briefly, a 2CS Cys-lite mutant (C247S and C412S) of N-terminally 6X-HIS tagged human SF-1 LBD comprising amino acids 218–461 of human SF-1 (21) which included a TEV site to remove the HIS tag was used for all studies. This 2CS mutant had been previously shown to improve crystal diffraction quality when compared with the WT SF-1 LBD (37). The SF-1 construct was expressed in BL21 *E. coli* DE3 bacteria and purified by immobilized metal affinity chromatography, and the HIS tag removed by overnight dialysis at 4°C under reducing conditions using 1 mg TEV per 10 mg SF-1 protein, desalted into buffer A (20 mM Hepes [7.5] with 2 mM CHAPS), and loaded onto a Capto-Q Hi-Trap ion exchange column, eluted isocratically with buffer A plus 300 mM NaCl, then desalted into 20 mM Hepes, all steps as previously described (21). To generate SF-1 LBD/PIP3 complexes, desalted SF-1 LBD in 20 mM Hepes (7.5) was incubated overnight at room temperature with 5:1 M excess of the phosphoinositide of interest (dioleoyl-PIP3 or dipalmitoyl-PIP3), nutating under nitrogen in borosilicate glass, the SF-1 LBD/PIP3 complexes were then separated from unexchanged SF-1 LBD using HiTrap-Q anion exchange chromatography (GE Biosciences) over a gradient of NaCl in 20 mM Hepes (7.5). The PIP3-bound SF-1 elutes at higher NaCl concentrations than SF-1 bound to ectopic bacterial phospholipid most likely because of the negative charges provided by the phosphates on PIP3 (21, 29). PIP3-bound fractions were pooled, concentrated to 25 mg/ml, then diluted to 10 mg/ml in buffer A, and used fresh for crystallization trials or frozen into aliquots at −80°C for use in functional peptide binding assays. Previous studies have shown freeze-thaw of purified human SF-1 LBD (2CS) does not detectably affect binding to any ligand tested (21, 29, 38).

### Phosphoinositide binding

Apparent K_d_ interaction data of phosphoinositides was determined on human apo-SF-1 LBD by a previously described native gel shift assay (21) to monitor equilibrium binding to each phosphoinositide. Briefly, apo-SF-1 ligand binding domain was generated using an on-column washout procedure to remove bound phospholipid, wherein pure SF-1 bound to dipalmitoyl PIP3 was loaded onto a Mono Q column and washed with 1.0 L of 20 mM Hepes (8.0), 1 mM EDTA with 2 mM CHAPS at 2 ml/min to remove all bound phospholipid from SF-1 by on-column dilution. The buffer was then changed into 20 mM Hepes (8.0), 1 mM EDTA to remove detergents, and apo-SF-1 eluted using a ammonium acetate gradient, pooled, protein concentration quantitated, and then used in the phosphoinositide binding assay. For the phosphoinositide binding assay, each phosphoinositide was resuspended in water, sonicated, diluted, and stored under nitrogen at 4°C. Phosphoinositides were always re-sonicated immediately before use in binding assays. Two microliters of serially diluted phosphoinositide were added to binding reactions of 25 μl total volume, containing 1 μM final concentration of apo-SF-1 LBD in 20 mM Hepes (8.0), 1 mM EDTA, and 10 mM ammonium acetate in 200 μl polypropylene PCR strip tubes. Binding reactions were incubated at 37°C for 1 h in a PCR thermocycler with a heated reaction cover, the reaction mixed with 3 μl native loading buffer (40% glycerol, 0.005% Ponceau S) and run on a 4% to 16% polyacrylamide Bis-Tris NativePage™ gel (Invitrogen). After fixation and silver staining (BioRad), gels were scanned, the phosphoinositide-shifted band quantitated using NIH image, and apparent K_d_ determined by nonlinear curve fit to a single-site binding model in GraphPad Prism. Data represent at least three independent replicates.

### Crystallization and structure determination

Crystallization trials were performed with human SF-1 LBD (218–461) 2CS “Cys-lite” mutant (C247S and C412S) complexed with dioleoyl PIP3(18:1/18:1) prepared as described above, and then complexed with 3-fold molar excess of a synthetic peptide representing the human PGC1α (accession NP_001317680.1) co-activator (H3N-EEPSLLKKLLLAPA-OH) purchased from New England Peptide. This peptide is identical to that used in co-crystallization of the dipalmitoyl PIP3-SF-1 structure (21), and both were added in a 3-fold molar excess in both crystallizations. The SF-1/PIP3 dioleoyl complexes were screened 1:1 with the PEG/Ion HT Screen (Hampton), using the TTP Labtech mosquito using the sitting-drop vapor diffusion method (100 nl screen; 100 nl SF-1/PIP3 at 10 mg/ml). Crystals formed at a final SF-1/PIP3 concentration of 5 mg/ml in 20% PEG 3350 and 0.2 M magnesium formate at 20°C. After 3 days, crystals were harvested and cryopreserved in 20% ethylene glycol and flash frozen in liquid nitrogen before X-ray analysis. Native diffraction data were collected at the Argonne National Laboratory Advanced Proton Source LS-CAT beamline 21-IDF. The data were indexed and scaled using HKL2000, diffraction data quality and twinning were assessed by Xtriage (no twinning was present), and the structure was determined using full-featured Phenix.PHASER-MR molecular replacement. Molecular replacement used PDB: 4QJR as the search model (human SF-1 LBD (218–461) 2CS Cys-lite mutant bound to dipalmitoyl PIP3 and the same PGC1α peptide). The structure was refined using Phenix.refine and Coot, validated using MolProbity tools, and uploaded to the protein data bank (PDB:7KHT).

### Fluorescence polarization coactivator binding assays

The same human SF-1 LBD (2CS) construct used for crystallization was complexed with dioleoyl PIP3 and subjected to anion exchange chromatography to separate PIP3-bound species from bacterial phospholipid-bound species. PIP3-bound SF-1 elutes during anion exchange chromatography at higher ionic strength than bacterial phospholipid-bound SF-1 because of the negatively charged phosphates on the PIP3 headgroup. The PIP3-bound and bacterial phospholipid–bound fractions were pooled and concentrated as above, then serially diluted into assay buffer (20 mM Hepes [7.5], 150 mM NaCl) in black-walled 384-well nonprotein binding plates, with 50 μM SF-1 LBD as the highest concentration and a constant 25 μL volume in each well. Reproducibility in the FP assay critically relies on using nonprotein binding plates. To each well, an equal volume of N-terminal 5Fam-labeled PGC1α co-activator peptide (5Fam-EEPSLLKKLLLAPA-OH) was added to a final concentration of 100 nM fluorescently labeled peptide (purchased from New England Peptide). After a 1-h incubation nutating at room temperature in the dark, fluorescence polarization was measured on the Biotek Neo Plate reader at the Vanderbilt High-throughput Screening Center. Data were fit to a standard one-site binding model in GraphPad Prism, and all data represent at least three independent replicates.

### Limited trypsin proteolytic sensitivity assays

Protease selection for limited digestion was carried out by adding elastase, thermolysin, proteinase K, or trypsin to SF-1 LBD in a protease:SF-1 LBD ratio of 1:500 (w/w), followed by incubation at 30 or 60 min and 10% SDS-PAGE followed by Coomassie stain. For determination of SF-1 proteolysis bound by PIP3(16:0/16:0) or PIP3(18:1/18:1), equal amounts of the purified SF-1 LBD used for crystallization without coactivator peptide were incubated with PIP3(16:0/16:0) or PIP3(18:1/18:1) at a protein:PIP3 molar ratio of 1:10, overnight at room temperature, a procedure determined in previous publications to result in complete PIP3 binding to SF-1 LBD. Limited proteolysis analysis was carried out by adding trypsin (Sigma) to SF1-PIP3 in a trypsin:SF-1 ratio of 1:500 (w/w), followed by incubation at 25°C for a 60 min time course. Aliquots of the reaction were taken just before trypsin addition (indicated as 0 min) and at 10 min, 30 min, and 60 min during the proteolysis reaction. Digestion was stopped by addition of SDS-PAGE loading dye and heating at 95°C for 5 min and frozen at −20°C. Samples were resolved on a 10% polyacrylamide gel and visualized with Coomassie blue staining. Gels were scanned, and the band intensities quantified using Image J (NIH).

### Lipid mass spectrometry

Human SF-1 LBD (33 μg) expressed and purified as above was treated overnight at room temperature with either DMSO vehicle or 10 μM RJW100 racemate to competitively displace phospholipids from the ligand binding pocket of SF-1, as previously reported (29, 38). These binding reactions were then desalted to separate SF-1 protein-bound lipids from unbound lipids and unbound RJW100. The SF-1 protein containing fraction after desalting was then precipitated by adding 200 μl of methanol to the protein solution, vortexed, and incubated overnight at −80°C. Precipitated protein was centrifuged at 4,200 rpm at 4°C to remove precipitate, and the phospholipid-containing supernatant was transferred to a new vial and dried under nitrogen at room temperature. The resultant phospholipid films were resuspended in 100 μl of 2-propanol with 0.1% formic acid: H_2_O (50:50) and analyzed by LC-MS using quadrupole time of flight (QTOF, 6560, Agilent) and tandem (MS/MS, 6470, Agilent) mass spectrometers coupled to LC (1290, Agilent). Multiple reaction monitoring (MRM) transitions for LC-MS/MS work are listed in supplemental Table C. LC gradients and mobile phase conditions for lipid separations were adapted from Reisdorph *et al*. (39), except mobile phase B consisted of isopropanol:acetonitrile (60:40) and 0.1% formic acid. LC-QTOF work verified the chemical formula for the PE lipids via accurate mass measurement (<2 ppm) shown in supplemental Table A. MRM LC-MS/MS data were acquired to quantify the relative abundance of lipids across the DMSO versus RJW100 treatment conditions as shown in supplemental Table B.

## Results

### Increasing acyl chain length and unsaturation alters phosphoinositide binding to SF-1

**Figure 1:**
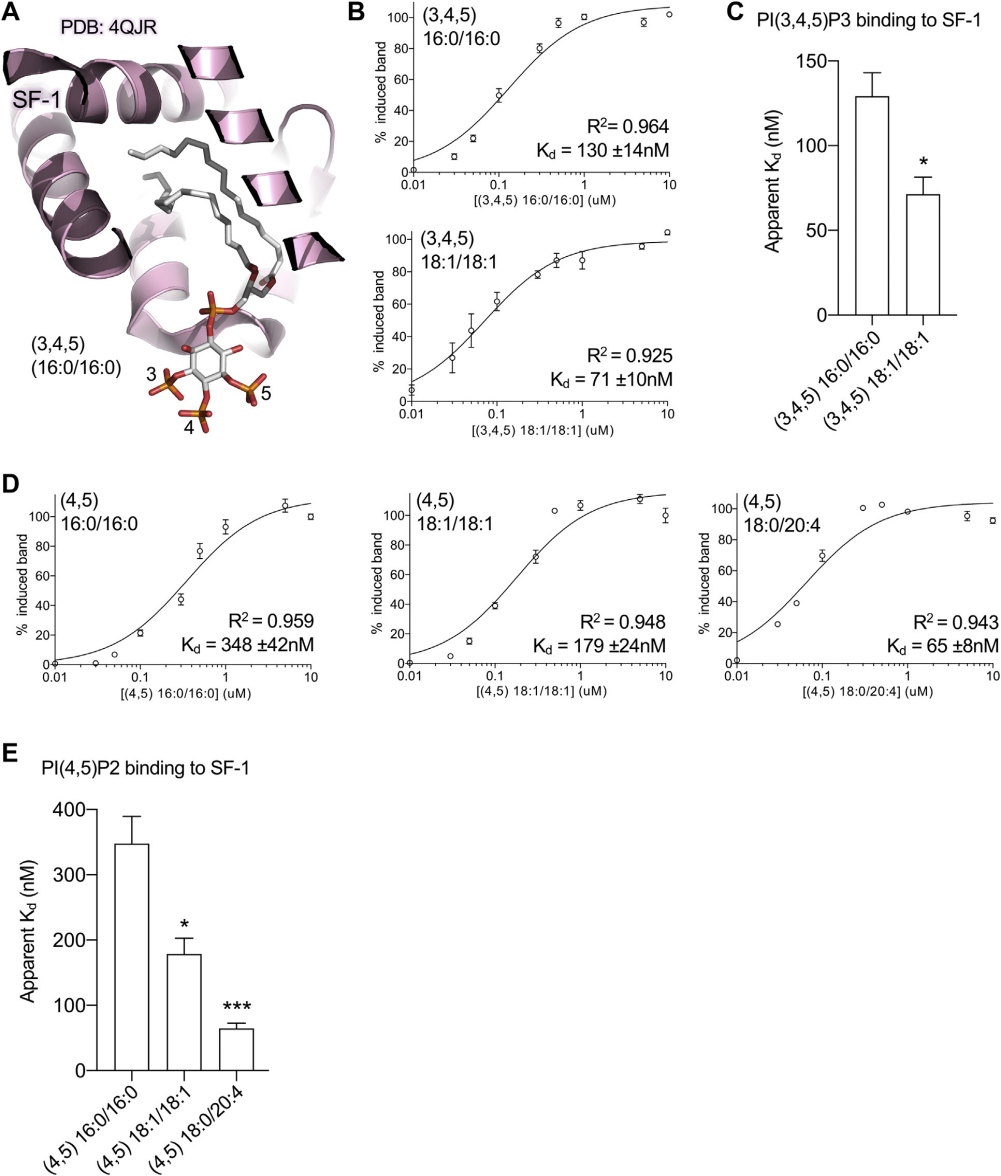
SF-1 binding to phosphoinositides is affected by the acyl chains. A: Cutaway view of previously published crystal structure of SF-1 bound to dipalmitoyl-PIP3 (PDB:4QJR) (21), showing the buried acyl chains. B: Apo-SF-1 ligand binding domain dissociation constants (K_d_) for interaction with PI(3,4,5)P3 with indicated acyl chains as measured by electromobility shift and fit to a 1-site binding model. C: Quantitation of (B), showing lower apparent K_d_ value for dioleoyl PIP3 (*t* test *P* = 0.0151, SEM). D: SF-1 interaction with PI(4,5,)P2 (PIP2) of indicated acyl chain lengths measured by electromobility shift and fit to a 1-site binding model as above. E: Quantitation of D comparing K_d_ values between PIP2 species when only the acyl chains are altered (*t*-tests ∗*P* = 0.0124 and ∗∗∗*P* = 0.0005, SEM). These data indicate phosphoinositide acyl chains can alter PIP2 and PIP3 apparent binding to SF-1. SF-1, steroidogenic factor-1.

Previous work established binding of phosphoinositides to SF-1 is modulated by specific headgroup phosphorylations at the 3 and 5 positions on the phosphatidylinositol ring, when acyl chain composition was held constant (21). Crystal structures of human SF-1 complexed with PIP3 explained this phenomenon, showing direct interactions between phosphorylations at the 3 and 5 positions of the PIP3 headgroup stabilize interaction with SF-1 (21). However, what effect the acyl chains might have on phosphoinositide binding to SF-1 (Fig. 1A) has not been addressed. Thus, we first asked if apparent binding of phosphoinositides to SF-1 LBD is affected by acyl chain length when the headgroup is held constant, using the same mobility shift assay we used in previous studies (21, 25, 29, 38). Phospholipid-less apo SF-1 generated using a previously reported washout procedure (21) was used to determine that dioleoyl (18:1/18:1) PIP3 bound to SF-1 LBD with a 3-fold lower apparent K_d_ than dipalmitoyl (16:0/16:0) PIP3 (71 ± 10 nM vs. 130 ± 14 nM, respectively) (Fig. 1B). These K_d_ values for the two PIP3 species are significantly different by *t* test (Fig. 1C, *P* = 0.0151). When holding the PI(4,5)P2 (PIP2) headgroup species constant while varying acyl chain length and unsaturation, the same pattern holds: The apparent K_d_ for PIP2 binding to SF-1 directly correlated in rank order with increasing PIP2 acyl chain length and degree of unsaturation: stearoyl/arachidonoyl (18:0/20:4) 65 ± 8 nM < dioleoyl (18:1/18:1) 179 ± 24 nM < dipalmitoyl (16:0/16:0) 348 ± 42 nM. These PIP2 binding apparent K_d_ values are significantly lower than dipalmitoyl (16:0/16:0) PIP2 values by *t* test (*P* = 0.0124 and *P* = 0.0005 respectively, Fig. 1E). These data suggest that along with previously studied influences of the phosphoinositide headgroup on binding to SF-1 (21, 40), the acyl chains of phosphoinositides can also participate in driving physical interaction with SF-1, suggesting the acyl chains might influence SF-1 structure and/or function.

### SF-1 LBD ectopically expressed in bacteria is bound by C16 and C18 acyl phospholipids

**Figure 2:**
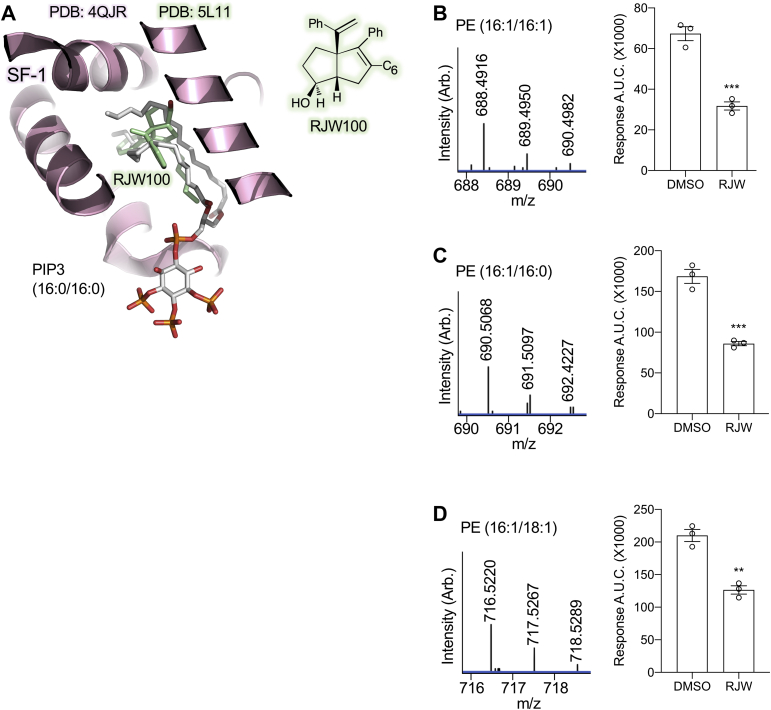
SF-1 associates with phospholipids containing C16 and C18 acyl chain lengths. A: Previously published crystal structure of SF-1 bound to dipalmitoyl-PIP3 (PDB:4QJR) (21), superposed with the RJW100 molecule from the structure of RJW100 bound to LRH-1, a close structural homolog of SF-1 (PDB:5L11) (47). Several studies have shown RJW100 binds to the same site as PIP3 in SF-1(29, 41, 44). B–D: QTOF mass spectra (mass error < 2 ppm, supplemental Table A) and LC-MS/MS quantification (supplemental Table B) to confirm association of SF-1 with PE species bearing (B) (16:1/16:1), (C) (16:1/16:0), and (D) (16:1/18:1) acyl chains. Bar graphs on right show quantitation of LC-MS/MS for each lipid species associated with SF-1 after displacement of phospholipids with either RJW100 or DMSO vehicle (supplemental Table B, *t* tests ∗∗∗*P* < 0.005; ∗∗*P* < 0.01, SEM). These data suggest SF-1 expressed in an ectopic system associates with phospholipids bearing C16 and C18 acyl chain lengths. LC-MS/MS, LC-tandem mass spectrometer; QTOF, quadrupole time of flight; SF-1, steroidogenic factor-1.

We next performed lipid mass spectrometry to identify acyl chain distributions of the most prominent phospholipids associated with recombinant human SF-1 LBD ectopically expressed in the DE3 bacterial cell expression system. To ensure phospholipids found by mass spectrometry were specifically associated in the lipid binding pocket of SF-1, we used the RJW100 synthetic small molecule to competitively displace phospholipids away from SF-1, followed by desalting to remove the displaced phospholipids before MS analysis. X-ray crystal structures of RJW100 complexed to the close SF-1 homolog LRH-1 suggest RJW100 binds to the same site as phospholipids (Fig. 2A), and previous studies have shown RJW100 competitively displaces phosphoinositides from SF-1 (38, 41). Thus, RJW100 treatment should only specifically decrease MS signal of phospholipids associated with the ligand binding pocket of SF-1. Purified SF-1 LBD samples treated with either DMSO vehicle or 10 μM RJW100 were desalted to remove free small molecules and analyzed by LC-QTOF mass spectrometry in positive ion mode. This revealed the bacterial phosphatidylethanolamine species associated with human SF-1 (supplemental Table A), confirming previous studies that identified PE associated with SF-1 purified from bacteria (15). Relative decrease of these lipids with RJW100 treatment using MRM on an LC-MS/MS was measured (supplemental Table B). We then targeted this analysis to three prominent acyl-chain species associated with SF-1 (16:0/16:1) (Fig. 2B), (16:1/16:1) (Fig. 2C), and (18:1/16:1) (Fig. 2D, supplemental Table B). These lipid mass spectrometry data suggest SF-1 ectopically expressed in bacteria associates with phospholipid species containing 16 or 18 acyl chain carbons and one unsaturation, although not to the exclusion of other acyl chain lengths or saturations. Because PIP3 has been proposed as a regulatory ligand for this nuclear receptor (21, 29, 40) and the PIP3(18:1/18:1) species is commercially available, we sought to determine how the acyl chains of PIP3 might regulate the SF-1 crystal structure.

### The crystal structure of SF-1 bound to dioleoyl (18:1/18:1) PIP3 adopts a typical nuclear receptor fold

**Figure 3:**
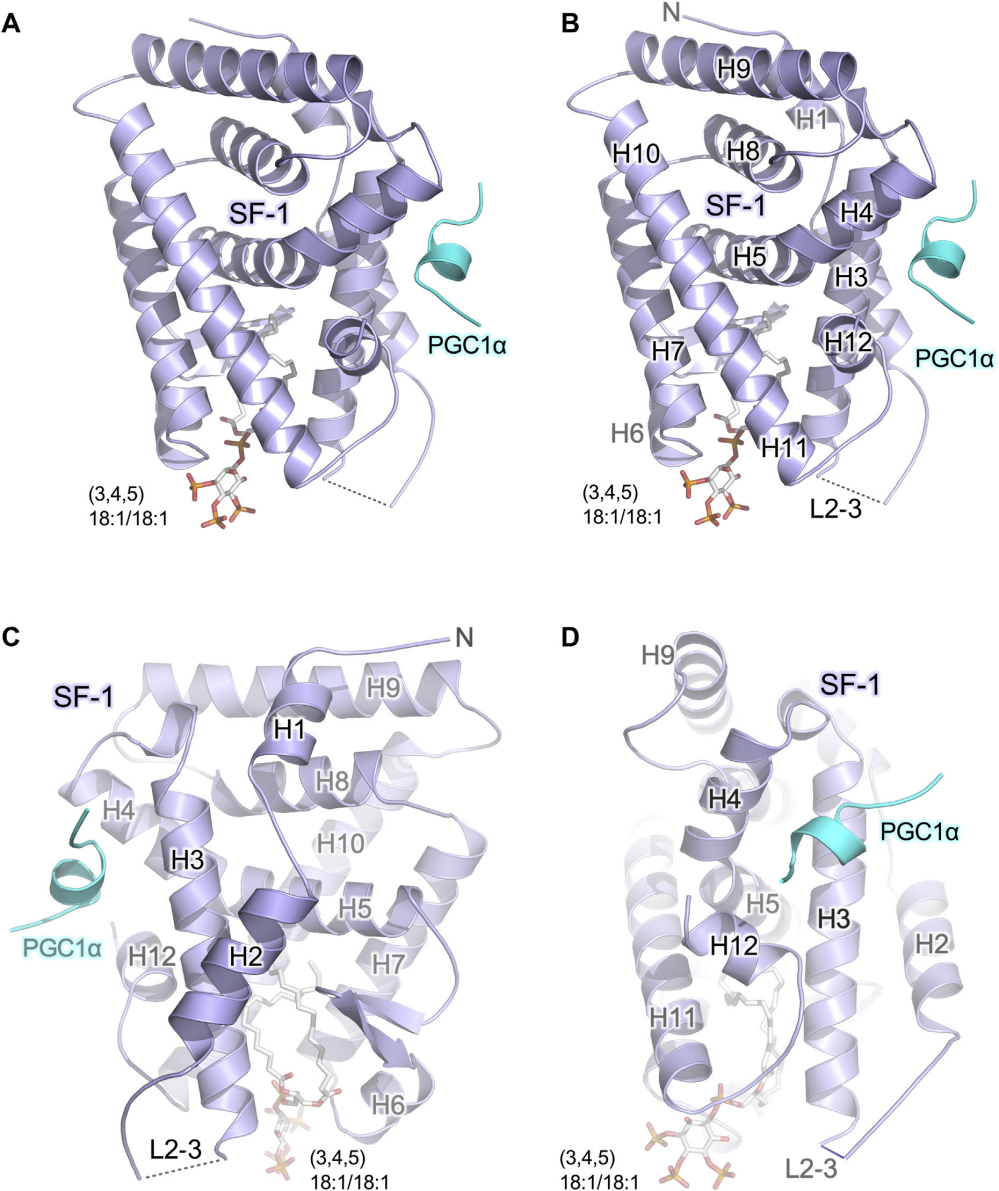
Crystal structure of SF-1 bound to dioleoyl (18:1/18:1) PIP3 adopts typical nuclear receptor 12-helix structure. A: Overall architecture of the co-crystal structure of human SF-1 ligand binding domain (residues 218–461) Cys-lite crystallization mutant (C247S and C412S, aids in diffraction (37)), colored in blue, bound to PGC1α coactivator peptide (EEPSLLKKLLLAPA) colored in cyan and PI(3,4,5)P3 (18:1/18:1) dioleoyl phospholipid shown sticks. B: Identical image as in (A), but with helices and loops labeled for orientation including the loop between helices 2–3 (L2-3), which was disordered in this structure. C: Rotation about the vertical axis as in B, to highlight well-ordered helices 1 and 2 common to NR5A subclass nuclear receptors (SF-1 and LRH-1). D: The co-activator binding/activation function 2 (AF2) site on SF-1 which interacts with PGC1α coactivator peptide (cyan) to control SF-1 transcriptional activity. These data indicate the crystal structure of human SF-1 bound to dioleoyl PIP3 adopts the gross nuclear receptor ligand binding domain (LBD) fold. PGC1α, peroxisome proliferator-activated receptor gamma coactivator 1-alpha; SF-1, steroidogenic factor-1.

We designed this experiment to match all aspects of our previously solved structure of human SF-1 bound to PIP3(16:0/16:0) (PDB:4QJR) (21) such that a comparison might be possible, despite the caveats of any crystallography experiment, expanded on below. To that end, we determined the 2.5 Å crystal structure of the same human SF-1 ligand binding domain construct complexed with the same 14-mer peptide (representing the PGC1α co-activator motif, EEPSLLKKLLLAPA) and dioleoyl PIP3(18:1/18:1) (supplemental Table D). Thus, all protein sequences were matched between the dioleoyl structure presented here and our previously published structure bound to dipalmitoyl PIP3 (21). In phosphoinositide mass spectrometry studies conducted in mammalian cells, the C36:2 MS peak can report dioleoyl species which is frequently observed in mammalian cells (42). As expected, the crystal structure of SF-1 bound to dioleoyl PIP3(18:1/18:1) adopts a 12-helical bundle structure (Fig. 3A, B) typical for nuclear receptor ligand binding domains, distributed into three layers, with fully ordered helices 1 and 2 (Fig. 3C) typical for the subclass of nuclear receptors which SF-1 belongs (NR5A) (15, 40, 43, 44). The 12 alpha helices end with the C-terminal helix 12, which helps form the site that binds the PGC1α coactivator peptide (Fig. 3D). This C-terminal helix 12 forms part of the activation function 2 (AF2) region, which is a part of the allosteric switch for lipid hormone-induced allosteric regulation of many nuclear receptors. In classic nuclear receptor activation (45, 46), lipid hormone binding alters the position of helix 12, in turn altering the capacity of the LBD to bind coregulator peptide. These data suggest the overall nuclear receptor fold of SF-1 bound to PIP3(18:1/18:1) is maintained.

### The acyl chains of dioleoyl PIP3 take a similar path through SF-1 as other co-crystallized acyl chains

**Figure 4:**
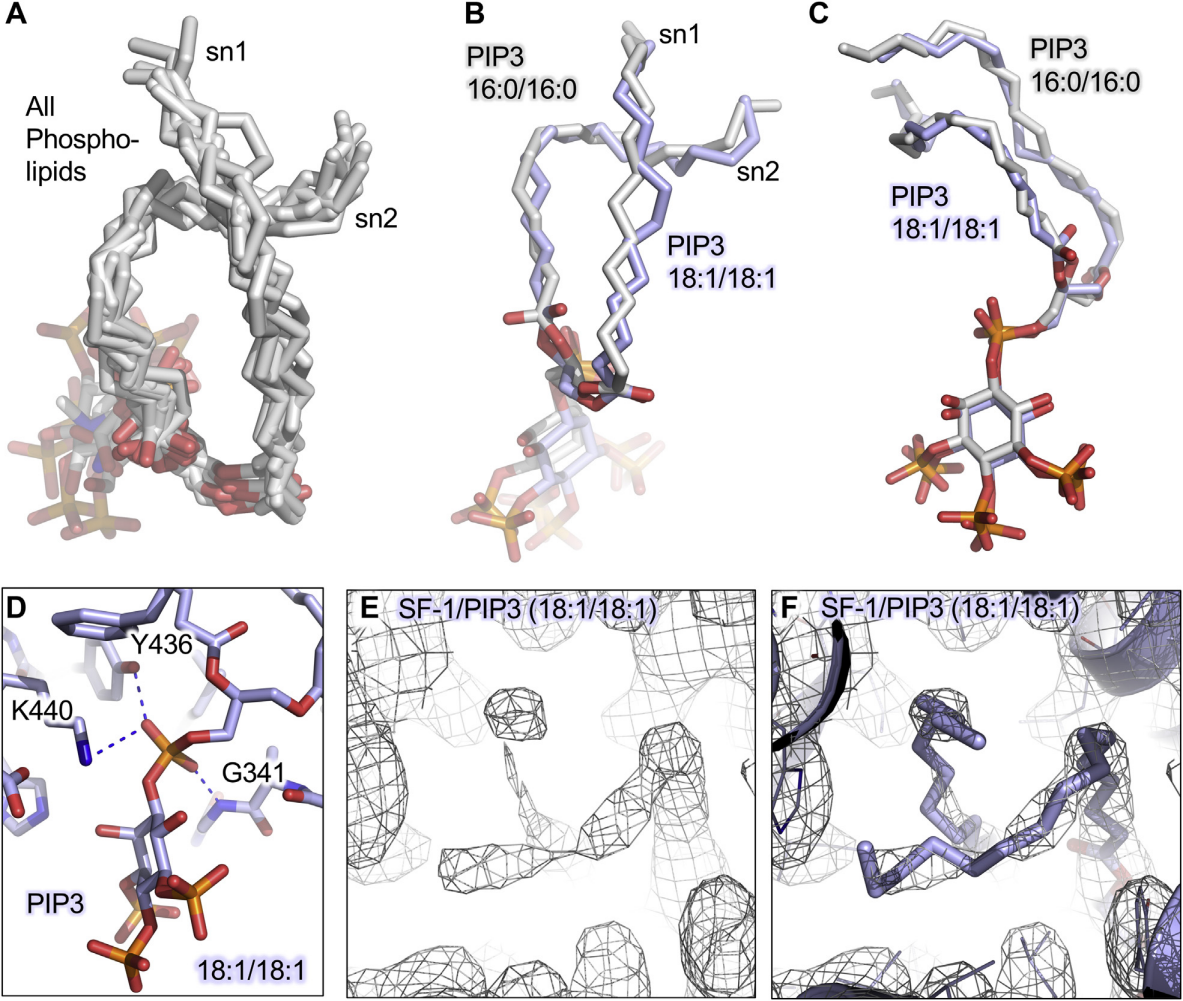
Acyl chains take a similar path through the hydrophobic core of SF-1 regardless of headgroup identity or acyl chain composition. A: Superposition of all phospholipids co-crystallized in seven previously published structures of SF-1 ligand binding domain (PDB ID: 1ZDT(51), 3F7D(16), 1YMT(15), 1YP0(33), 1YOW(15), 4QK4(21), and 4QJR(21)). B and C: Superposition of dioleoyl PIP3(18:1/18:1) colored in blue reported here versus the previously reported structure 4QJR of dipalmitoyl PIP3(16:0/16:0) (21), both co-crystallized with the same human SF-1 sequence, in the same crystallographic space group and with the same PGC1α coactivator peptide. D: Close-up of the sn3 glycero-phosphate at the 1-position of the inositol ring of PIP3, showing coordination of the phosphate by indicated human SF-1 residues. E: Electron density map contoured at 1.0 sigma shown as mesh with F modeled dioleoyl acyl chains of PIP3 shown as sticks, residing deep in the hydrophobic core of human SF-1 protein, colored as blue cartoon. These data indicate the acyl chains of phospholipids travel a similar path through SF-1, regardless of headgroup or acyl chain composition. PGC1α, peroxisome proliferator-activated receptor gamma coactivator 1-alpha; SF-1, steroidogenic factor-1.

The unsaturation of the dioleoyl acyl chains of PIP3(18:1/18:1) could change the path of the acyl chains through the core of SF-1 compared with the fully saturated dipalmitoyl acyl chains. However, the acyl chains of all phospholipids co-crystallized with SF-1 (15, 16, 21, 32, 37) take an indistinguishable path through the hydrophobic core of SF-1 as shown by superposition of the phospholipids from the seven crystal structures in the protein database (PDB) of both mouse and human SF-1 (Fig. 4A). Each of these structures was complexed with a different combination of peptide, phospholipid and/or SF-1 sequence (mouse or human), yet all show a very similar path taken by phospholipids bound by SF-1 (Fig. 4A). Still, no two structures have been “matched” such that the only variable between them is acyl chain identity. Comparing our matched dioleoyl versus dipalmitoyl structures confirms the acyl chains in both structures follow similar paths, with the sn1 acyl chain crossing over and on top of the sn2 chain (Fig. 4B) and the PIP3 headgroups similarly positioned (Fig. 4C). The position of the PIP3 headgroups in these structures is in sharp contrast to the “flipped” orientation that occurred in the PIP2 dipalmitoyl-bound structure of SF-1 (21). It is important to note that the phosphate at the sn3 position of PIP3 is tightly coordinated at one three-dimensional position relative to SF-1 by several conserved SF-1 amino acids (K440, Y436, G341, Fig. 4D) (15, 16). This coordination is thought to somewhat restrain the position of the phospho-glycerol at a single site in SF-1, potentially restraining the position of the “first” alpha carbon on each acyl chain (15, 16). Indeed, the PIP3 headgroup is in a similar position in the dipalmitoyl and dioleoyl-bound structures (Fig. 4C), and density for the last two omega carbons of the dioleoyl acyl chains was poorly defined, suggesting the last two carbons on both acyl chains are disordered in these crystals (Fig. 4E, F). Together, these data suggest the acyl chains of dioleoyl and dipalmitoyl PIP3 follow a similar path through the hydrophobic core of SF-1.

### SF-1 crystal structures induced by dioleoyl versus dipalmitoyl PIP3 are not identical

We then sought to compare how the crystal structure of SF-1 bound to dioleoyl PIP3(18:1/18:1) was altered compared with our previously reported structure of SF-1 bound to dipalmitoyl PIP3(16:0/16:0). Again, these complexes were crystallized with identical sequences and components such that the only designed differences between the complexes were the acyl chains of PIP3. Differences in the two crystals could also be because of unintended crystallographic factors such as changes in pH, altered cryopreservation, or any myriad of other factors. However, the dioleoyl PIP3 crystals fortuitously formed in the same space group as dipalmitoyl crystals (P4_1_2_1_2), crystallization pH was the same (pH 7.5), cryopreservation conditions were the same (20% ethylene glycol), and all constructs/peptides used in both crystals were identical (21). Further, the most important difference observed in the structures was validated using solution functional studies presented below. Thus, the changes observed in these two crystals are most consistent with acyl chain–induced changes to SF-1 protein structure, as determined by X-ray crystallography.

### Dioleoyl PIP3 induces structural disorder in the loop between helix 2 and helix 3

**Figure 5:**
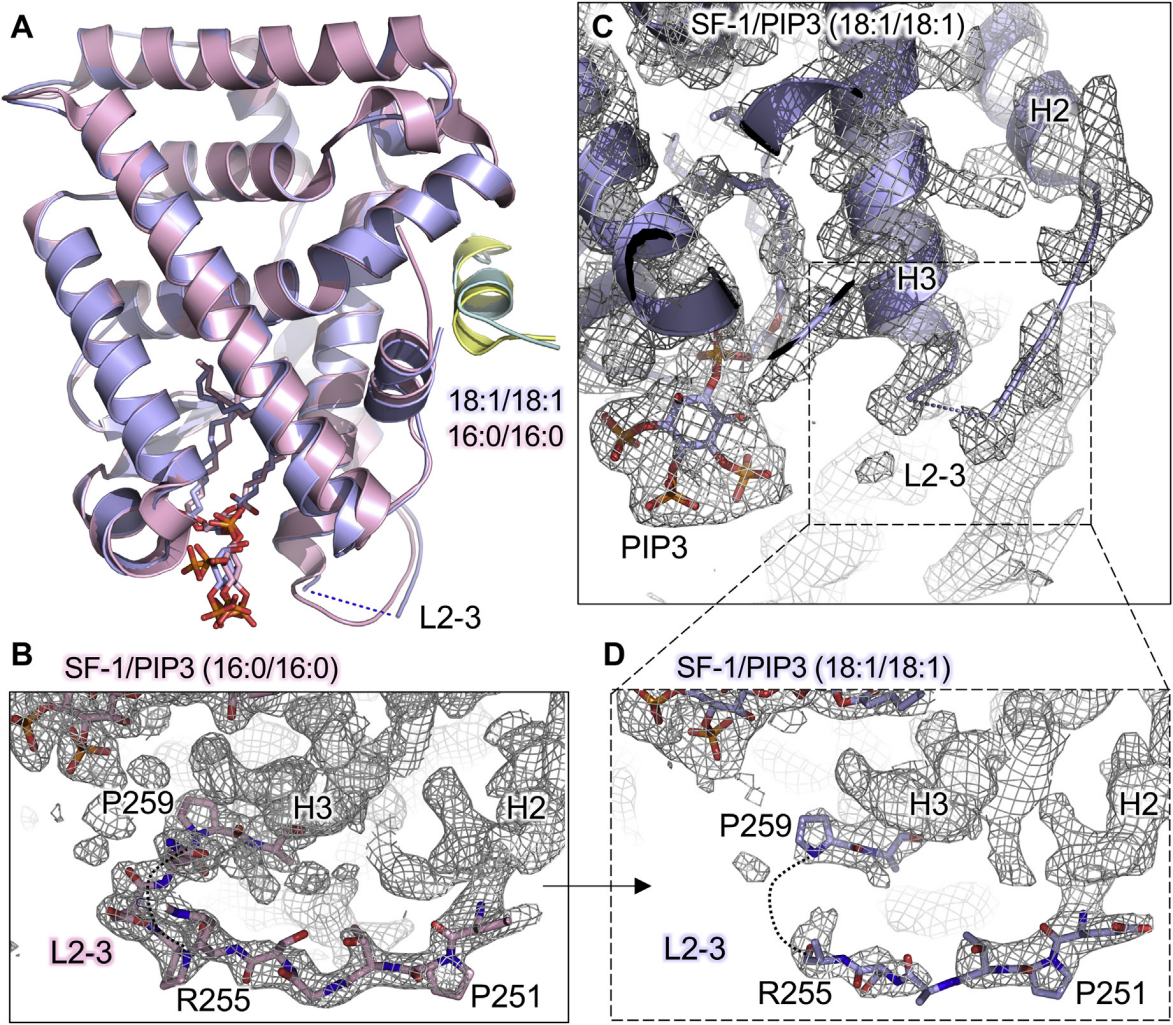
The SF-1 loop between helices 2–3 is disordered in the PIP3(18:1/18:1)-bound crystal structure. A: Superposition of SF-1 structures bound by dioleoyl (blue) versus dipalmitoyl (pink) and identical PGC1α coactivator peptide (green peptide bound to dioleoyl, yellow peptide bound to dipalmitoyl), showing lack of 2–3 loop residues in the dioleoyl-bound structure. B: Electron density map of dioleoyl PIP3-bound SF-1 (SF-1/PIP3 (18:1/18:1)) contoured at 1.0 sigma showing electron density at the site of the loop between helices 2–3 (L2-3). C: Close-up of L2-3 showing fit of R255 and P259 residues to available density, contoured to 1.0 sigma. D: Close-up of electron density in the same L2-3 region of the dipalmitoyl PIP3-bound structure also contoured at 1.0 sigma, showing fit of L2-3 residues as well as side chains. These data indicate dioleoyl PIP3 induces disorder in SF-1 L2-3 when compared with dipalmitoyl PIP3. PGC1α, peroxisome proliferator-activated receptor gamma coactivator 1-alpha; SF-1, steroidogenic factor-1.

In comparing the two PIP3-bound structures, the overall RMSD of SF-1 bound to dioleoyl versus dipalmitoyl PIP3 is 0.319 Å over 189 C-alpha atoms (Fig. 5A), suggesting grossly similar conformations. SF-1 residues in the loop between helix 2 and helix 3 (2–3 loop) have been shown to be clinically important for SF-1 function in human patients (33, 34, 35, 36). However, it has not been previously possible to attribute changes in 2–3 loop order directly to acyl chain length, as other differences were present in those co-crystal structures (15, 16, 21, 32, 37). The 2–3 loop was well-ordered in the dipalmitoyl-bound structure (Fig. 5B), but dioleoyl PIP3 induced disorder in the 2–3 loop when compared with the well-matched dipalmitoyl PIP3-bound structure. Comparing electron density in this 2–3 loop region in our structure here shows less density available for model fitting in the dioleoyl-bound structure (Fig. 5C, D). The 2–3 loop is the site of polymorphisms found in patients presenting with adrenal insufficiency (35), ovarian insufficiency (36), male infertility (34), and disorders of sexual development (33), all attributed to loss of SF-1 function in these patients (supplemental Table E). Two such polymorphisms are R255L and R255C, the R255 residue was the last residue in the 2–3 loop that could be assigned with main chain density in the PIP3(18:1/18:1) bound structure but still lacks sufficient density to assign the guanidinium side chain (Fig. 5D). The 2–3 loop density is clearly visible for all main chain residues in the dipalmitoyl PIP3-bound structure of SF-1, including side chain density for R255 (Fig. 5B). Together, the data suggest that when crystallized, the 2–3 loop of SF-1 is less well ordered when bound to dioleoyl PIP3 compared with dipalmitoyl PIP3.

### Dioleoyl PIP3 induces structural disorder in the coactivator peptide region

**Figure 6:**
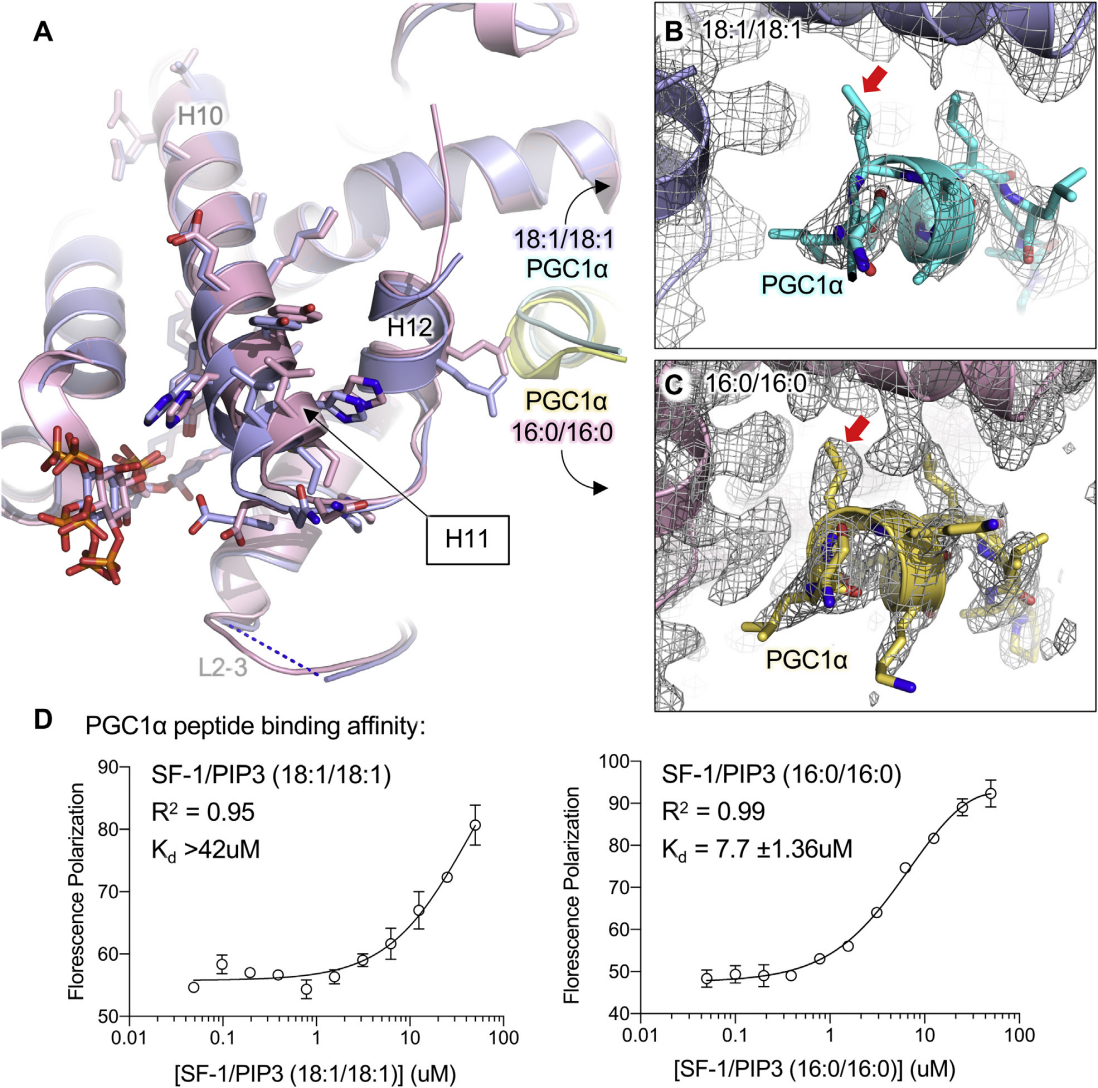
The acyl chains of dioleoyl PIP3(18:1/18:1) regulate PGC1α peptide interaction with SF-1. A: Superposition of the region around helix 11 of the dioleoyl (blue) versus dipalmitoyl (pink) PIP3-bound SF-1 structures, highlighting shift of H11 away from H12 in the dioleoyl-bound structure. B: Electron density contoured to 1.0 sigma of the PGC1α peptide region in the dioleoyl-bound structure, red arrow indicates electron density around LxxLL motif leucine residue important for interaction between nuclear receptor and co-activator peptide. C: Same site as in B but in the dipalmitoyl-PIP3 bound structure, again with electron density contoured to 1.0 sigma, and the red arrow indicating density around the same leucine residue in the LxxLL motif of the PGC1α peptide. D: Fluorescence polarization binding of fluorophore-labeled PGC1α peptide to SF-1 ligand binding domain complexed with either dioleoyl PIP3 (dissociation constant K_d_ > 42 μM) or dipalmitoyl PIP3 (apparent K_d_ = 7.7 ± 1.4 μM). These data indicate dioleoyl PIP3 induces both structural (panels A–C) and functional (panel D) changes to SF-1, in vitro. PGC1α, peroxisome proliferator-activated receptor gamma coactivator 1-alpha; SF-1, steroidogenic factor-1.

The most dramatic difference between the dioleoyl versus dipalmitoyl PIP3-bound SF-1 structures is observed in the functionally critical coactivator peptide (PGC1α), which binds SF-1 at the AF2 region, composed partly of helix 12 at the C-terminus of the protein. Helix 12 is well established to mediate nuclear receptor transcriptional activity by forming the ligand-regulated binding site for coactivators. In crystallographic studies of nuclear receptors, large coactivator proteins are often represented by a short peptide rather than the full-length protein, to permit crystallization (20, 46). Here, we observe that helix 11 has a clear shift away from helix 12 in the dioleoyl-bound structure of SF-1 (Fig. 6A), although helix 10 at the N-terminus of helix 11 is almost indistinguishable between the two structures. Dioleoyl-PIP3 induced more disorder in the electron density representing the PGC1α peptide (Fig. 6B) when compared with the identical peptide in the dipalmitoyl PIP3-bound structure (Fig. 6C). During refinement, it became clear the fit of the PGC1α peptide to available electron density was very poor, resulting in substantial penalties in real space Z-scores for the N-terminal and C-terminal residues of the peptide. These RSRZ penalties were accepted such that more of the peptide could be included in the deposited coordinates for completeness and for comparison with other sequence-matched structures. Together, the data suggest dioleoyl PIP3 induces a shift of H11 away from H12 in the crystal, as well as more disorder in PGC1α peptide bound to SF-1, when compared with the well-matched dipalmitoyl PIP3 crystal structure.

### Dioleoyl PIP3 functionally represses SF-1 binding to PGC1α coactivator peptide in solution

To test if the disordered state observed in the dioleoyl PIP3-bound SF-1 crystal translates to a functional change in SF-1 activity free in solution, we used solution-based fluorescence polarization to determine SF-1 binding to a fluorophore-labeled PGC1α peptide. Note these assays measure the affinity of coactivator peptide for SF-1 LBD as induced by phospholipid, not affinity of phospholipid for SF-1 as measured in Fig. 1. We previously used surface plasmon resonance to determine the K_d_ of an unlabeled PGC1α coactivator peptide for SF-1/PIP3(16:0/16:0) as 6.55 ± 0.4 μM, and the K_d_ of peptide for SF-1 bound to co-purifying bacterial phospholipid as 8.8 ± 0.8 μM (21). Here, we show the K_d_ of a fluorophore-labeled PGC1α peptide for SF-1 bound to bacterial phospholipid is similar between the assays (Fig. 6D, supplemental Table F). Dioleoyl PIP3(18:1/18:1) induces SF-1 affinity for the PGC1α peptide >42 μM, at least a 5-fold poorer affinity for SF-1 than induced by PIP3(16:0/16:0) (K_d_ = 7.7 ± 1.4 μM) (21) (supplemental Table F). These in vitro functional assays show dioleoyl PIP3 induces weaker SF-1 binding to the PGC1α coactivator peptide than that induced by dipalmitoyl PIP3 or induced by ectopically associated bacterial phospholipid (supplemental Table F). These data are consistent with the poor crystallographic order observed in the PGC1α coactivator peptide in the PIP3(18:1/18:1)-bound SF-1 crystal structure (Fig. 6B, C) and with increased trypsin protease sensitivity of SF-1 bound to PIP3(18:1/18:1) (supplemental Figs S1–3). Together, these data suggest the acyl chains of dioleoyl PIP3 induce a conformation of SF-1 that is less optimal for interaction with the PGC1α transcriptional coactivator peptide. The studies presented here provide the first structural and functional consequences for SF-1 driven by PIP3 acyl-chain composition.

## Discussion

Before this study, to query how the phospholipid acyl chains might regulate SF-1 structure (15, 16, 21, 32, 37), one might have considered comparing the two PDB structures 1YOW (SF-1 bound to TIF2 coactivator peptide and phospholipid PE [16:1/18:1]) (15) versus 1YP0 (SF-1 bound to SHP corepressor peptide and PE [16:0/16:0]) (32), although the co-crystallized peptides in these structures are not matched so structural differences may be induced by peptide, not lipid. Still, in comparing these structures, the loop between helix 2 and helix 3 (2–3 loop) is disordered in the (16:1/18:1) bound structure but ordered in the (16:0/16:0) structure, consistent with observations made here (Fig. 5). In this study, we establish in the context of well-matched crystals (peptides, protein sequences, and phospholipid headgroups all identical), the acyl chains alone are indeed capable of regulating the crystal structure of human SF-1 ligand binding domain (Fig. 5). It will be interesting to determine using solution-based structural techniques such as NMR spectroscopy, if noncrystallographic SF-1 protein dynamics are similarly altered by the lipid acyl chains.

Changes in the chemical nature of the acyl chains might be predicted to influence the acyl chain path through the hydrophobic core of SF-1. A survey of phospholipid-bound crystal structures of SF-1 (15, 16, 21, 32, 37) suggests the acyl chains follow an indistinguishable path through the hydrophobic core of SF-1, regardless of acyl chain length or unsaturation (Fig. 4). This observation holds true for the dioleoyl and dipalmitoyl PIP3-headgroup and peptide-matched structures compared here. Thus, the data from many different structures all suggest the overall path the acyl chains take through the hydrophobic core of SF-1 is not changed by phospholipid headgroup composition, degree of unsaturation, or acyl chain length, although the paths of acyl chains with other lengths/unsaturation remains to be determined.

The phosphate at the sn3 position common to glycerophospholipids had been previously established to be coordinated at a single position by conserved amino acids (15). This coordination is thought to restrain the position of the glycero-moiety within SF-1, thus restraining the position of the alpha carbon on each acyl chain, consistent with the co-crystal structures of SF-1 bound to phospholipids reported in the protein database (Fig. 4A). If the alpha carbon were positionally restrained, acyl chain lengthening might be expected to alter the position of the omega carbon, more so than altering the position of the phospholipid headgroup relative to the SF-1 protein. Indeed, the PIP3 headgroup positions were similar in both the dipalmitoyl and dioleoyl-bound structures (Fig. 4C), although a slight shift of the headgroup away from helix 12 is observed in the dioleoyl structure (Fig. 6A). However, no large differences were observed in the helices or side chain positions close to the omega carbons of the acyl chains. Thus, although functional changes are induced by the different acyl chains (Fig. 6), how structural changes induced by the acyl chains are allosterically translated to the peptide binding (AF2) region remain unclear. It is therefore likely structural techniques better suited to examining dynamics will be required to distinguish the allosteric paths used by dipalmitoyl versus dioleoyl PIP3 to regulate SF-1 structure.

Data presented here are most consistent with the acyl chains of phosphoinositides controlling the ordered state of the loop between helices 2 and 3 (2–3 loop), providing context to further our understanding of how polymorphisms in this region might decrease SF-1 activity in patients. Coding changes to residues in the 2–3 loop associate with clinical diseases generally accepted to be driven by loss of SF-1 function in vivo. Four of these polymorphisms in the *NR5A1* locus that reside in the coding sequence of the 2–3 loop associate with clinical diseases (supplemental Table E), coding for A260V associated with 46,XX ovotesticular disorders of sex development (33), D257N associated with male infertility (34), R255L associated with adrenal insufficiency (35), and R255C associated with primary ovarian insufficiency (36). Data presented here show the phospholipid acyl chains control crystallographic order in the 2–3 loop where these polymorphisms reside, consistent with these polymorphisms inducing loss of SF-1 function potentially by decoupling phospholipid binding from the structural order of the 2–3 loop. These new data also suggest the chemical nature of the acyl chains may participate even more than the phosphoinositide headgroup in controlling 2–3 loop order, as in both dipalmitoyl PIP2 and PIP3 structures, the 2–3 loops were well-ordered (21), whereas in the dioleoyl PIP3 structure presented here, the 2–3 loop was highly disordered (Fig. 5B–D). Of course, without in-solution confirmation of these effects on the 2–3 loop, any differences could be simple in vitro artifacts; thus, more studies are required. However, we previously showed the R255L mutant in the 2–3 loop does not acquire phospholipid ligand as well as WT SF-1, indicating this mutation associates with defective phospholipid transfer/acquisition (16). Taken together, the data currently suggest a connection between structural order in the 2–3 loop and SF-1 activity, although more direct tests of this hypothesis are required.

Our data are also consistent with the 2–3 loop playing a role in full-length SF-1 structural regulation. Not only does the overall transcriptional activity of the R255L mutant decrease in cells but also this mutant does not detectably bind DNA oligos in electromobility shift assays (35). Those data suggest some form of interdomain communication must occur between the SF-1 and DNA-binding domain (DBD), because the R255 amino acid is located in the LBD but changes function of the DBD. We recently developed a structural model describing LBD–DBD communication for full-length LRH-1 (*NR5A2*), a close homolog of SF-1 (*NR5A1*) (44). That model of LRH-1 shows LBD helix 2 is a key site for an interaction between the LBD and DBD, and disrupting the interdomain interaction on the LBD side alters DNA-binding on the DBD side of the interface (44). Although three-dimensional homology between full-length SF-1 and LRH-1 proteins is unclear, it is tempting to speculate that 2–3 loop disorder induced by acyl chain composition could translate acyl chain occupancy information from the LBD to the DBD, altering SF-1 DNA-binding and transactivation, that the R255L mutant fails to shift DNA oligos is consistent with a model in which the stability of the LBD–DBD interaction is regulated by acyl-chain occupancy of the LBD in WT SF-1, and that the R255L mutant may lose function, in part, by preventing full LBD–DBD communication via the 2–3 loop. Any future work on how acyl chain composition might regulate LBD–DBD interdomain communication will first require a structural model of full-length SF-1, which does not currently exist.

Together, the studies presented here show the acyl chains of PIP3 regulate the structure and function of human SF-1 ligand-binding domain in vitro, for the first time directly addressing how phosphoinositide acyl-chain composition regulates SF-1. These data identify a need to systematically establish structure–activity relationships for specific acyl chains, potentially explaining how SF-1 polymorphisms contribute to adrenal insufficiency, ovarian insufficiency, and other disorders of sexual development in human patients.

## Data availability

Complete crystal structure coordinates and structure factor files are on deposit at the Protein Data Bank (PDB: 7KHT), which can be found online at https://www.rcsb.org/structure/unreleased/7KHT. All other data are contained within the manuscript.

## Supplemental Data

This article contains supplemental data.

## Conflict of interest

The authors declare that they have no conflicts of interest with the contents of this article.
