# Supplementary material for: The acyl chains of phosphoinositide PIP3 alter the structure and function of nuclear receptor steroidogenic factor-1
Source: J Lipid Res. 2021 Apr 29;62:100081. doi: 10.1016/j.jlr.2021.100081 (PMC8178125; doi:10.1016/j.jlr.2021.100081)
Supplement: Supplemental Figures S1 to S3 and supplemental Tables A to F [file mmc1.pdf]

Bryant, et. al. Supplemental Fig 1:

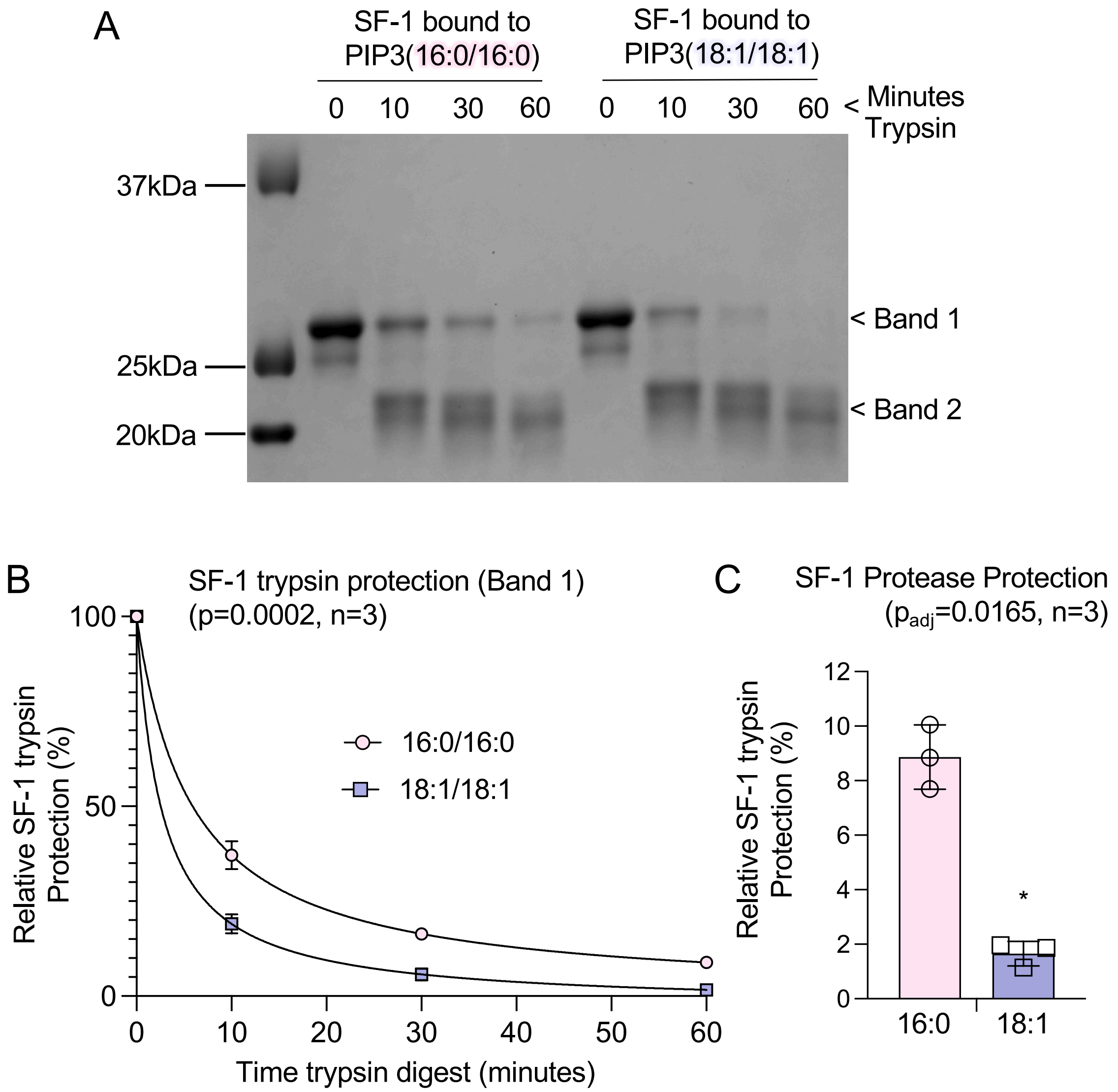

Supplemental Figure S1: *SF-1 bound to dioleoyl PIP3(18:1/18:1) is more sensitive to limited protease digestion than SF-1 bound to dipalmitoyl PIP3(16:0/16:0).* **A**. Coomassie-stained SDS-PAGE of trypsin-digested SF-1 LBD bound to indicated PIP3 species, trypsin treated over a 60-minute time course, reactions boiled in SDS-buffer and run on a 10% gel. **B.** Relative quantitation of band 1 expressed as a percentage of the total signal from bands 1 and 2 in each lane, quantified using ImageJ (p=0.0002 by two-way ANOVA, n=3). **C.** Bar graph comparing band 1 of SF-1 LBD bound to either PIP3(16:0/16:0) indicated as 16:0, or PIP3(18:1/18:1) indicated as 18:1, after 60 minutes of trypsin digest (**p_{adj}= 0.0165, Sidak's multiple comparisons test, n=3). These data suggest PIP3(18:1/18:1) induces a more trypsin-sensitive conformation in SF-1 ligand binding domain, compared to PIP3(16:0/16:0).

Bryant, et. al. Supplemental Fig 2:

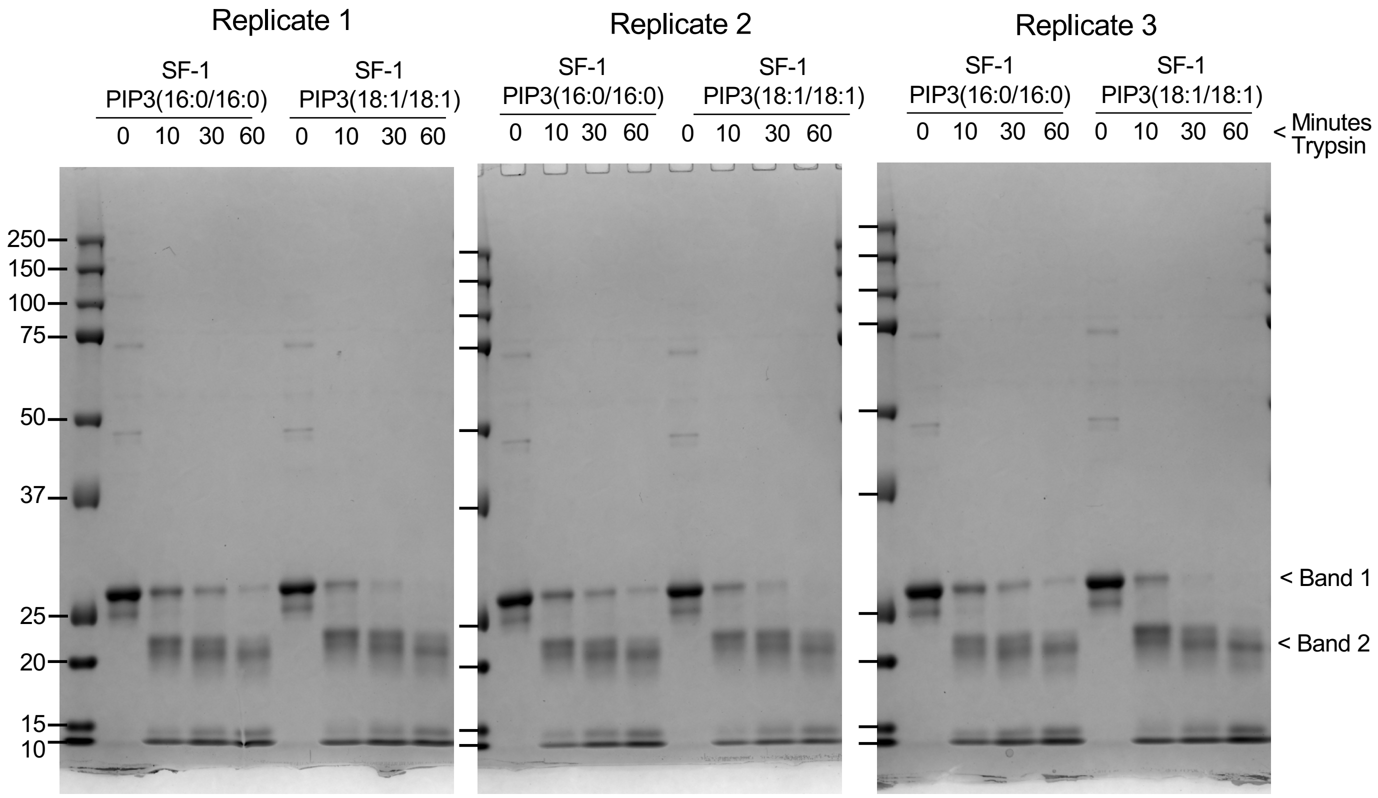

Supplemental Figure S2: Coomassie-stained SDS-PAGE of each of three limited trypsin protease digestions replicates, showing the whole lanes and marker migration (kDa), quantitated in Figure S1B and S1C, as indicated. Replicate 1 is the representative data shown in Fig S1, panel A.

Bryant, et. al. Supplemental Fig 3:

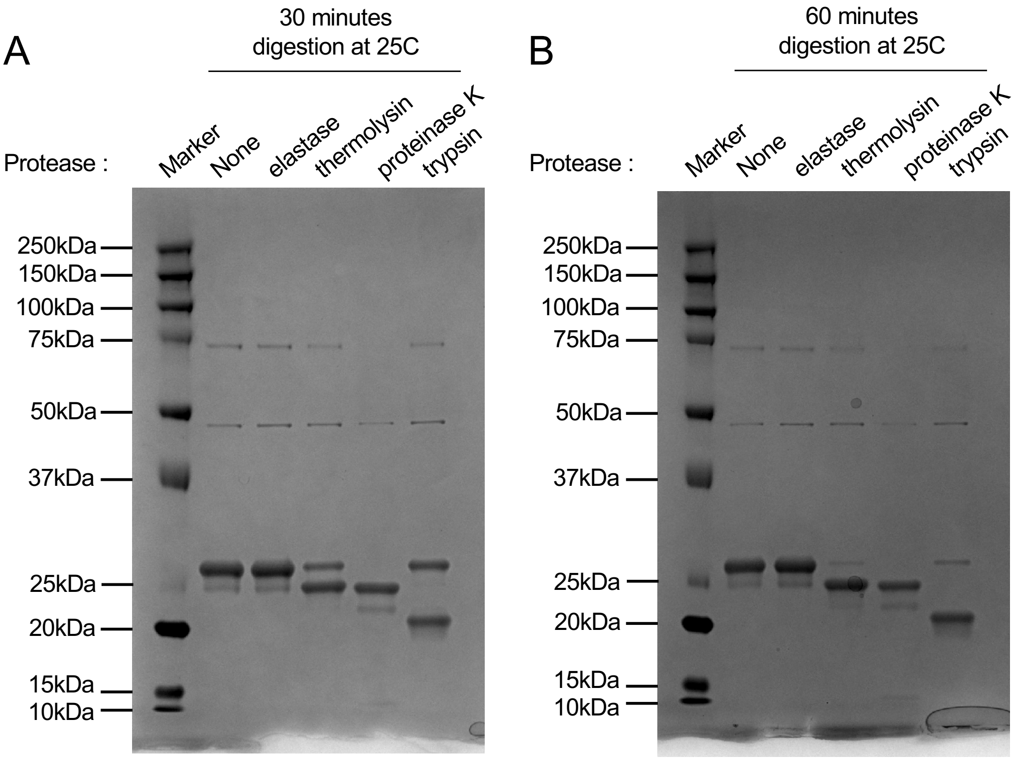

Supplemental Figure S3: Coomassie-stained SDS-PAGE of indicated SF-1 LBD limited protease digestion experiments for **A.** 30 minutes and **B**. 60 minutes, at a protease:SF-1 LBD ratio of 1:500 at 25C. Band 1 in undigested LRH-1 LBD lanes migrates at the expected weight for the SF-1 ligand binding domain of approximately 28kDa. For the trypsin digestion, the lower band 2 migrates at approximately 20kDa.

Bryant, et. al. Supplemental Tables A-C:

Supplemental Table A. Chemical formula verification of SF-1 bound lipids with LC-QTOF.

	Formula	Formula	Monoisotopic Mass		Mass Error
			Theoretical	Measured	
Lipid	Neutral [M]	[M+H]	[M+H] (m/z)	[M+H](m/z)	ppm
PE (18:1/16:1)	$C_{39}H_{74}NO_8P$	$C_{39}H_{75}NO_8P$	716.5230	716.5220	1.396
PE (16:1/16:0)	$C_{37}H_{72}NO_8P$	$C_{37}H_{73}NO_8P$	690.5074	690.5068	0.869
PE (16:1/16:1)	$C_{37}H_{70}NO_8P$	$C_{37}H_{71}NO_8P$	688.4917	688.4916	0.145

Supplemental Table B. Relative response of PE lipids from RJW100 treatment with LC-MS/MS.

	Response 688.49		Response 690.51		Response 716.52	
	RJW	DMSO	RJW	DMSO	RJW	DMSO
	31860	69875	81149	182039	114778	224451
	35231	71587	89894	171086	136790	213060
	28215	60579	86912	152521	127982	192735
Avg:	31769	67347	85985	168549	126517	210082
StDev:	3509	5923	4445	14922	11079	16066
% CV:	11	9	5	9	9	8
% Change:	-	52.83	-	48.98	-	39.78

Supplemental Table C. LC-MS/MS MRM parameters.

Lipid	Precursor Ion (m/z)	Product Ion (m/z)	Transition	MS1/MS2 Resolution	Dwell (ms)	Fragmentor (V)	Collision Energy (V)	Cell Acc. (V)	Polarity
PE (18:1/16:1)	716.5	575.43	Quantifier	Unit	40	150	20	5	Positive
	716.5	265.25	Qualifier	Unit	40	150	40	5	Positive
PE (16:1/16:0)	690.5	549.42	Quantifier	Unit	40	150	20	5	Positive
	690.5	237.22	Qualifier	Unit	40	150	40	5	Positive
PE (16:1/16:1)	688.5	547.4	Quantifier	Unit	40	150	20	5	Positive
	688.5	237.22	Qualifier	Unit	40	150	40	5	Positive

Bryant, et. al. Supplemental Table D:

Supplemental Table D. Data Collection and Refinement statistics.

**PDB ID**	**7KHT**
Resolution Range	38.54 - 2.504 (2.594 - 2.504)
Space Group	P 41 21 2
Unit Cell	65.349 65.349 139.676 90 90 90
Total Reflections	21999 (2114)
Unique Reflections	11000 (1053)
Multiplicity	2.0 (2.0)
Completeness (%)	98.38 (98.78)
Mean I/sigma(I)	21.78 (2.60)
Wilson B-factor	70.45
R-merge	0.00850 (0.2889)
R-meas	0.01202 (0.4086)
CC1/2	1 (0.912)
CC*	1 (0.977)
Reflections used in refinement	10847 (1052)
Reflections used for R-free	510 (40)
R-work	0.22 (0.36)
R-free	0.25 (0.36)
CC(work)	0.962 (0.778)
CC(free)	0.976 (0.835)
Number of non-hydrogen atoms	2044
macromolecules	1945
ligands	66
solvent	33
Protein Residues	247
RMS(bonds)	0.010
RMS(angles)	1.30
Ramachandran favored (%)	98.34
Ramachandran allowed (%)	1.66
Ramachandran outliers (%)	0.00
Rotamer outliers (%)	0.47
Clashscore	6.51
Average B-factor	93.64
macromolecules	93.66
ligands	95.64
solvent	88.35
Number of TLS groups	10

Statistics for the highest-resolution shell shown in parentheses.

Bryant, et. al. Supplemental Tables E-F:

Supplemental Table E. Clinically relevant *NR5A1* polymorphisms in the 2-3 loop.

Coding *NR5A1* polymorphism	Clinical presentation in patients	Reference
p.Ala260Val (A260V)	Presented heterozygous with 46,XX ovo-testicular disorder of sexual development.	(35)
p.Asp257Asn (D257N)	Presented nonsense with male-factor infertility.	(36)
p.Arg255Leu (R255L)	Presented as XX female with transcriptionally inactive SF-1, normal ovarian function but adrenal insufficiency (37). Decreased phosphoinositide binding *in vitro* (16).	(37, 16)
p.Arg255Cys (R255C)	Presented as XX female heterozygous with decreased transactivation of *CYP11A1* and *AMH* SF-1 target promoters.	(38)

Supplemental Table F. PGC1α peptide binding to SF-1.

SF-1/Lipid Complex	PGC1α apparent K_d	Reference
SF-1/PIP3 (18:1/18:1)	>42uM	This study
SF-1/PIP3 (16:0/16:0)	7.7 ±1.4uM	This study
SF-1/PIP3 (16:0/16:0)	6.6 ±0.4uM	(21)
SF-1/Bacterial Phospholipid	6.8 ±2.0uM	This study
SF-1/Bacterial Phospholipid	8.8 ±0.8uM	(21)
